# Characterizing Cognitive Deficits and Dementia in an Aging Urban Population in India

**DOI:** 10.1155/2012/673849

**Published:** 2012-06-27

**Authors:** G. Nair, K. Van Dyk, U. Shah, D. P. Purohit, C. Pinto, A. B. Shah, H. Grossman, D. Perl, V. Ganwir, S. Shanker, M. Sano

**Affiliations:** ^1^Neurology Department, T. N. Medical College/BYL Nair Charitable Hospital, Mumbai 400008, India; ^2^JJP VA Medical Center, Bronx, NY 10468, USA; ^3^Psychiatry Department, Mount Sinai School of Medicine, New York, NY 10029, USA; ^4^Neurology Department, KEM Hospital and Seth GS Medical College, Mumbai 400012, India; ^5^Pathology Department, Mount Sinai School of Medicine, New York, NY 10029, USA; ^6^Psychiatry Department, T. N. Medical College/BYL Nair Charitable Hospital, Mumbai 400008, India

## Abstract

Rapid rise in the population of older adults in India will lead to the need for increased health care services related to diagnosis, management, and long-term care for those with dementia and cognitive impairment. A direct approach for service provision through memory clinics can be an effective, successful, and sustaining means of delivering specialized health care services. We have established a memory clinic in Mumbai, India by employing the diverse clinical skills available in Indian academic institutions, diagnostic and research expertise of clinicians and psychologists, and the support of the U.S. National Institutes of Health. Our project involved recruitment of patients, clinical and neuropsychological assessment, and standardized diagnostic procedures, demonstrating the feasibility of using research methods to develop a memory clinic. In this paper, we describe the development of a community-based memory clinic in urban India, including linguistic and cultural factors and present detailed results, including diagnostic characterization, on 194 subjects with various stages of cognitive deficits. Our findings support the feasibility of developing a memory clinic in a public hospital and successful use of research diagnostic criteria to categorize cognitive deficits observed in this population, which may be used to inform the development of other such clinics.

## 1. Introduction

The mental health of aging persons in India is receiving growing attention as the country is projected to be the home of one of the world's largest populations of elders [1]. By 2025, the number of persons in India over 60 is projected to reach over 150 million [2]. In light of this rapidly increasing population, dementia and cognitive impairment are expected to increase as well. A comprehensive review of mental health conditions in India across the lifespan summarizes the growing problem of dementia [3]. Prevalence rates of dementia in India over the past two decades ranged from approximately 1.4% among those 65 years and older [4] to 3.5% among those 60 years and older [5] in rural settings; in urban settings, prevalence rates were reported as 2.44% among those 65 years and older [6]. Recently, however, crude prevalence rates of dementia in India have been reported as high as 10.6% in persons 65 and older living in rural areas and 7.5% for those in urban areas [7].

Early detection of cognitive impairment and dementia is critical to its management and will require developing the capacity to efficiently evaluate and manage occurrence of disease [8]. The establishment of specialized memory clinics can provide the capacity to identify and treat dementia in the population and can also act as a resource from which information about disease and its management can be shared with other practitioners and throughout the community. Evaluations must be able to provide differential diagnoses of specific subtypes of dementia and impairment and be sensitive enough to capture staging of disease severity in order to direct appropriate interventions. Among developing countries, as elsewhere, Alzheimer's disease and vascular dementia are the two most common types of dementia observed [9]. The assessment of mild cognitive impairment (MCI) may be more challenging in these populations due to the lack of normative data across the breadth of socioeconomic strata that exists in a country as large as India. Several research efforts have reported assessing dementia subtypes in India with commonly used measures. The 10/66 Dementia Research Group has successfully employed dementia evaluations including neuropsychological tests in cross cultural contexts in order to inform diagnoses of Alzheimer's type dementia based on DSM-IV criteria [10]. In addition, standardized measures have been used to characterize Alzheimer's disease, vascular dementia, and dementia with Lewy bodies [11, 12]. Neuropsychological evaluations in this population have been used to diagnose mild cognitive impairment [13] in some settings. These are encouraging efforts for the utility of commonly used dementia assessment methods in developing countries such as India and support the development of standardized dementia assessments in memory clinics to evaluate and characterize cognitive impairment.

Nonetheless, there are few reports of memory clinics in India, particularly in public hospitals in medical school settings where the patient population is diverse, and models of clinical services need to accommodate the challenges of multiple linguistic and cultural factors as well as educational and economic extremes. In this paper, we report the results of integrating commonly used research methods in dementia evaluation with a diverse sample of older adults seen at a public hospital. We also provide our experiences in dealing with the challenges faced in assessing cognitive disorders in this population. 

Our research team established a memory clinic under the Cognitive Disorders in the Elderly Joint Indo-US Dementia Research Project in Mumbai, funded by an NIH/NIA/FIC grant. This clinic was designed to facilitate clinical and neuropsychological dementia evaluations and offer participants treatment options, as well as house dementia studies and clinical trials. Clinical researchers from Mount Sinai School of Medicine's Alzheimer Disease Research Center (MSSM ADRC) collaborated with clinicians in the Topiwala National Medical College/Nair Charitable Hospital, a state-run public hospital in Mumbai, India, in order to plan, coordinate, and execute the establishment of the clinic in the hospital. The purpose of this preliminary paper is to describe the development of a memory clinic in Nair Charitable Hospital, including the outreach process, the evaluation methods including linguistic and cultural considerations, training the clinical team, and developing consensus diagnostic procedures, and describe the clinical population seen at the clinic. 

## 2. Methods

### 2.1. Outreach Process

The clinic was established as a center for individuals with memory and cognitive problems, which offered evaluation, treatment, and management including education for families and caregivers. In order to raise awareness about the utility of the services offered at the memory clinic, outreach efforts focused on education about dementia and cognitive disorders and included description of the possible personal and social impact of these disorders. The initial outreach for the clinic was made through the departments of Psychiatry and Neurology at Nair Hospital which are the home departments of the local Principal Investigators of the research project. The broader medical community was then engaged and informed of the clinic through special lectures, seminars, and workshops sponsored by Nair and open to the medical and aging care communities throughout Mumbai. 

### 2.2. Evaluations

The data reported here was collected from patients seen in the memory clinic who underwent the informed consent process for research as approved by the Nair Charitable Hospital and Topiwala National Medical College Ethics Committee. Informed consent procedures for those who may not have been able to give consent followed the ethical guidelines of the Nair Hospital Ethics Committee, including obtaining a signature from a proxy. While the clinic would provide the clinical evaluation without consent, the consent allowed for summarizing and reporting data from the clinical evaluation. Virtually all who were asked to participate agreed to consent. Participants were then administered a standardized clinical evaluation and battery of neuropsychological tests. These instruments were modeled after the National Alzheimer's Coordinating Center (NACC) standardized Uniform Data Set (UDS) evaluation [14] (which is available at the following URL http://www.alz.washington.edu/WEB/forms-uds.html) and the MSSM ADRC's clinical core assessment (available upon request from the author, M. Sano). 

The evaluation included a semistructured interview with both the patient and an informant in order to provide an accurate and comprehensive account of symptoms. In addition to basic demographic information, a semistructured assessment of the presenting cognitive complaints, a history of symptoms, a quantified functional assessment, and measure of instrumental and basic activities of daily living are collected. Psychiatric, medical, and family history of dementia and cognitive loss were recorded. Laboratory tests were ordered to rule out medical illnesses such as B12 deficiency, thyroid illness, and acute infection when needed. Medical comorbidities were recorded including diabetes, hypertension, thyroid dysfunction, and coronary and pulmonary disease. Global mental status and disease severity were assessed using the Mini-Mental State Examination (MMSE) [15] or Hindi version of the MMSE [16] and clinical dementia rating scale (CDR) [17]. Mood and psychiatric disorders were assessed by patient and informant (usually family)—reported history and with psychiatric and behavioral symptom scales (Neuropsychiatric Inventory [18] and Geriatric Depression Scale [19]). An adapted blessed functional activity scale [20] assessed activities of daily living. Neurological examination included the clinician assessment of cognition, motor, and sensory function and a standardized assessment of extrapyramidal symptoms (EPS), based on the UPDRS [21]. All participants were advised to undergo clinical imaging (CT or MRI) however, since the procedure was not included in this study, it is not available for all cases. When available, scans were reviewed by the clinic's neurologist, and imaging results were recorded and discussed during diagnostic consensus conferences.

### 2.3. Training the Clinical Team

The clinical team included local neurologists, psychologists, and a social worker. A study neurologist conducted the semistructured clinical interviews and physical examinations. Study neurologists were trained in administering the semistructured interview through tutorial and observation of experienced ADRC dementia clinicians. Master's level psychologists (the minimum educational level required to practice psychology in India) administered a neuropsychological assessment (results not reported here). Standardized training procedures for conducting the neuropsychological assessment included viewing a standardized training DVD, observing neuropsychological assessments performed by the senior psychologist at the memory clinic, and administering neuropsychological assessments under the supervision of a neuropsychologist. Deidentified copies of the neuropsychological scoring protocols were sent to the MSSM ADRC psychometrician who reviewed them for administration and scoring errors and provided feedback. All staff received training and certification where available to conduct standardized measures such as mental status (MMSE [15] or HMSE [16]) and disease severity (CDR) [17]. We used standardized training techniques which have been demonstrated to yield reliable results [22]. A social worker facilitated clinical visits and clinical followup, including visiting the home as needed. 

### 2.4. Diagnosis

Research diagnostic categories and methods of assignment were modeled after the NACC Uniform Data Set [14] and the MSSM ADRC. Diagnostic worksheets, designed to be completed by a clinician, were created to ensure classification was made according to published criteria. All cases received a consensus diagnosis either at an internal (Nair Hospital clinicians and staff only) or joint (Nair Hospital and MSSM clinicians and staff) consensus conference. Joint conferences were held during site visits and over Skype conference calls, and all consensus conferences were attended by at least one clinician and one psychologist. Specific diagnostic categories were based on standard research criteria: dementia of the Alzheimer's type, as outlined in the DSM-IV [23]; probable and possible Alzheimer's disease (AD) [24]; probable and possible Ischemic Vascular Dementia (VaD) [25]; mixed dementia, defined as the presence of ischemic vascular dementia and another systemic or brain disorder causing dementia [25]; dementia with Lewy bodies (DLBs) [26–28]; frontotemporal dementia (FTD) [29]; mild cognitive impairment (MCI) both amnestic and nonamnestic types [30]; other disorders, such as psychiatric disorders, were diagnosed when standard criteria was available. 

### 2.5. Linguistic and Cultural Factors

Careful consideration of linguistic and cultural factors was required prior to applying the clinical research and assessment methodology described above in this population. One of the first challenges was identifying age or date of birth. Since some participants, particularly those of low literacy, were not able to specifically identify their age or date of birth, methods of approximation were used. Such methodology included first asking the participant or informant to spontaneously recall major historical events and temporally relate them to personal milestones during childhood and adulthood. A prepared list of historical events was then reviewed with the participant or informant who was asked again to relate them to personal milestones. Similar methodology has been previously described [31]. 

Interview and assessment were completed in one of four languages, English, Hindi, Marathi, and Gujarati (among the most common languages spoken in Mumbai) depending on the language the patient was proficient in. All examiners were proficient in all four languages. However, some cases required use of an interpreter (other clinical staff in the hospital). Separate interviews were offered with individual informants or family members in order eliminate possible discomfort from discussing the patient's cognitive or functional difficulties in the presence of the patient. Additionally, staff members of both genders were available to discuss what may be viewed as socially inappropriate behaviors with informants and family members.

The high rate of illiteracy and low education in this population required consideration and adaptation of evaluation methods. The HMSE was used for those who were of low literacy as items were adapted for illiterate rural Indian populations [16]. Several assessment items required further explanation (e.g., providing an example of what sentence is) or concrete, real-world framing (e.g., subtracting rupees for serial subtractions). Additionally, demonstrations were offered for alternating movement instructions during the neurological exam. 

### 2.6. Data Analysis

This paper summarizes demographic and clinical features using descriptive statistics (mean, SD, range, and frequency) for the cohort recruited between the years 2006 and 2010. Diagnostic categories were enumerated and demographics and medical history are provided for major diagnostic groups. The HMSE has a maximum score of 31, whereas the maximum MMSE is 30. In order to combine scores across participants into one variable, the HMSE was converted to a percent correct which was then calculated out of 30 and was combined with MMSE scores into one adjusted MMSE variable (this method has not been validated). Demographic and medical comorbidity variables were examined with one-way ANOVA (age and years of education) or chi-square or Fisher's exact test (gender, medical comorbidities) to compare differences among the three most common major diagnostic groups, AD, VaD, and MCI. For the purposes of these analyses, MCI amnestic and nonamnestic groups were combined into one MCI group [32]. All analyses were performed at the *P* = .05 level and computed using SPSS 17.0 or 19.0 software. 

## 3. Results

During the recruitment period, 212 participants were evaluated for cognitive complaint by Nair Hospital's memory clinic. The majority of these cases were referred by clinicians from the Nair Neurology or Psychiatry Departments. Among those who came to the clinic with a cognitive complaint, 18 did not receive a diagnosis: four were evaluated too soon after acquiring an ischemic stroke to make an accurate diagnosis; the clinical evaluation was not able to be completed for 13 cases; and in one case, the data was deemed insufficient to make a diagnosis. Therefore, this paper is based on data from 194 clinical cases. CT or MRI imaging results were available on 135 of these 194 cases and available to the members of the consensus conference when these cases were reviewed. Of the 194 cases, date of birth was approximated in 103 cases.

### 3.1. Demographics and Medical Conditions

Demographic features and medical conditions of the sample are summarized in Table 1. Notably, there is a high percentage of males (64.4%) in this group. The medical conditions assessed are also outlined in Table 1 (data on history of hypertension and diabetes was not available on 3 of the clinical cases, and data on the rest of the conditions was not available on 4 clinical cases; percents are of cases for which data was available). Hypertension was the most common medical condition in the sample, observed in 45.9% of cases. Diabetes and stroke were also present in approximately 20% of cases. A high number (35.3%) of the clinical samples with completed neurological exams (*n* = 187) were observed to have at least one extrapyramidal symptom (e.g., resting tremor or rigidity). Primary language was available for 192 cases, of these Marathi was the most common (52.1%), followed by Hindi (17.7%), Gujarati (10.4%), and others (19.8%). 

### 3.2. Presenting Complaint

Among those cases seen in the clinic, memory change was the most common presenting complaint and was present in 66.5% of cases. The next two most common presenting complaints were personality change (13.5%) and change in behavior (12.4%). Few participants complained of change in performance (8.3%), language, (6.7%), disorientation (5.2%), depressed mood (2.1%), or psychosis (5.2%). In twenty five clinical cases, more than one presenting complaint was recorded. 

### 3.3. Diagnostic Assignment

Of the 194 cases that were referred to the clinic for a cognitive complaint and received a diagnosis, 65.5% had a diagnosis of dementia (see Table 2). Among those with dementia, 45.7% had AD (probable or possible), 22% had VaD (probable or possible), 15% had mixed dementia (coincident with VaD, progressive dementias in this group included AD, DLB, and Parkinson's disease-related dementia), and 11% had FTD. The remaining approximately 6% of dementia cases had other diagnoses including dementia due to alcohol abuse or neurotrauma, dementia with Lewy bodies, vitamin B-12 deficiency, or Parkinson's disease-related dementia.

Of the nondementia clinical cases, approximately 64.2% were diagnosed as MCI (amnestic *n* = 33, and nonamnestic *n* = 10), 28.3% had a primary psychiatric disorder (e.g., depression, schizophrenia, psychosis, etc.), and 7.5% were not impaired.

### 3.4. Dementia Diagnoses

The three most common dementia diagnoses were Alzheimer's disease, vascular dementia, and mixed dementia. Significant differences (*F*(2, 102) = 5.16,  *P* < .01) in age were found between the VaD ($\overset{-}{x}=60.21\pm 10.97$) and AD ($\overset{-}{x}=67.64\pm 9.56$) groups, but not between the mixed dementia group ($\overset{-}{x}=65.05\pm 10.01$) and either other group. In addition, there was a significantly higher frequency of females in the AD group (50%) and mixed dementia groups (36.8%) than the VaD group (7.1%; *P* < .001; and *P* = .02, resp.) however, no differences were observed between the AD and mixed dementia groups (*P* = .42). Hypertension was reported more frequently in the VaD group (74.1%; data unavailable for one case) than in the AD group (41.4%; *P* < .01) however, no differences were found between the mixed dementia group (68.4%) and AD or VaD groups. The groups did not differ on any other demographic (i.e., years of education, percent illiterate) variables. 

### 3.5. MCI, AD, and VaD

AD and MCI participants were found to be of similar age however, the VaD group was significantly younger than both the AD and MCI groups (see Table 3). Performance was compared on global measures of cognition, function, and psychiatric symptoms between the AD, MCI, and VaD groups (complete data for each measure was unavailable for some cases in each of the diagnostic groups). While there were no significant differences observed between the AD and VaD groups on these measures, overall significant differences were observed between both dementia groups, AD and VaD, and the MCI group on all measures, indicating worse cognition, worse functioning, and more psychiatric symptoms in the dementia groups. There were no differences observed on the geriatric depression scale between any groups. 

## 4. Discussion

This work described the challenges of establishing a memory clinic in an urban Indian community and provides evidence of the feasibility of evaluating a diverse patient population. Key elements of the clinic that contributed to its success included organized outreach efforts, standardized evaluations, training for the clinical team, and consensus-based diagnostic processes. 

While outreach efforts were comprehensive, the majority of referrals were from knowledgeable neurologists or psychiatrists seeking a comprehensive dementia evaluation for an existing cognitive complaint. It was also likely that in most cases the cognitive complaint was brought to the clinician only after there was interference with activities of daily living. Additionally, patients and family members who came to the memory clinic displayed little knowledge or understanding of dementia or cognitive impairment as a medical entity. This highlights several important issues in this population: first, despite receiving referrals predominantly from specialized clinicians with expertise in dementia, there was considerable uptake of the memory clinic's services indicating the substantial need for such memory clinics in this community. Second, there remains a great need for education about dementia and cognitive symptoms among general medical settings, communities, and the public at large, beginning with raising awareness about dementia as a distinct medical entity.

Cultural considerations were key to engaging the community. Since this is a culture that holds high respect for elders, discussing what may be viewed as shortcomings (i.e., cognitive symptoms) can be viewed as a sign of disrespect. Thus, separate, private interviews with individual informants and family members were an important provision in order to collect unhindered information about true deficits. Additionally, same-gendered interviewers also facilitated more forthcoming discussion of behavioral symptoms that may be viewed as socially inappropriate (e.g., hypersexuality, abusive behaviors). In addition, some of the items of the basic and instrumental activities of daily living were not culturally relevant measures of functioning. For example, the item that measures the participant's ability to eat rates eating with one's hands as impaired, but among this population eating with one's hands is quite common and culturally normal. In such cases, the physician assessing the participant used other items to determine a change from a previous level of functioning. The spoken version of many Indian languages lacks a term for dementia, and “memory impairment” is frequently used to describe a wide range of deficits; thus, exhaustive interviews with informants were often required to obtain a clear understanding of the presentation. The very low literacy of our sample also presented a challenge. While illiteracy in India is high [33], our sample had higher rate of illiteracy than the city of Mumbai [34], which may be a result of recruitment through a state-run hospital. 

The distribution of our dementia diagnoses is similar to other reports from India [6, 11, 35] and reports of memory clinics from other developing countries [36, 37], with the most common diagnosis of Alzheimer's disease, followed by vascular dementia. Alzheimer's disease is recognized as the most common type of dementia in both developed [38] as well as in developing countries [9]. This complements the known neuropathology which is similar in urban Indian samples and urban samples from the US [39].

As a whole, there were more males than females in this sample. However, the gender distribution is similar to that of western cohorts when diagnostic category is taken into account [40]. In particular, the high proportion of males in the MCI group is comparable to that seen in western samples [41], and a similar pattern in the VaD group has been observed in other reports from memory clinics in India [12]. It is unclear if this represents a difference in medical use pattern or differential awareness or sensitivity to cognitive complaints by gender. 

Among the comorbidities assessed in this population, hypertension surfaced as the most common medical comorbidity in both the clinical and comparison groups. Detected hypertension is generally treated in this cohort; however, this data was not available for this publication since initial evaluations often depended on report of medication use without confirmation from other service providers or from more knowledgeable family members. The prevalence of hypertension in the Indian population is receiving increased attention, as it may become a serious health concern among the nation's population of older adults [42, 43]. Hypertension has been found to be a significantly higher risk factor for vascular dementia than AD in other urban Indian samples [13]; in our sample, hypertension was more frequently found in the VaD and mixed dementia groups than in the AD group. In light of the high frequency of hypertension observed here and in other studies, and of the known contribution to vascular disease, future studies might determine its contribution to the rate of cognitive decline in neurodegenerative illness. 

As expected, the cognitive, functional, and psychiatric measures successfully characterized the dementia, AD and VaD, and MCI groups. The AD and VaD groups were observed to be more cognitivelyand functionally impaired than the MCI group, and the AD and VaD groups alsohad more neuropsychiatric symptoms than the MCI group. All groups endorsed a comparable, subthreshold number of items on a depression scale. 

We note that the age of this cohort is younger than many identified in western countries. Yet it is not dissimilar from ages reported from other clinical cohorts in India with dementia [11, 12]. This is probably not surprising in a setting where illiteracy is high and education is low [35]. Educational achievement is rapidly changing in India as is life expectancy, and with this, we may expect to see a shift in the demographic characteristics of the clinical population. Additionally, in our sample, the MCI group was found to be more highly educated than the AD and VaD groups. This discrepancy may reflect a higher self-awareness in the MCI group, prompting them to be clinically examined at an early indication of some impairment. These findings highlight the need to include sensitive measures to capture the sometimes subtle cognitive and functional changes in MCI across educational levels. In addition, clinical outreach and educational efforts should be attentive to the wide range of education in this older population and how that may impact initial cognitive complaint. 

Our sample is one of convenience and may not represent the population as a whole, but it may be representative of those who seek treatment at a public hospital. However, an advantage of assessing a clinical population is that the cases are referred with clear complaint and often functional deficits. When clinical history can document decline in cognition and function, the need to depend solely on normative neuropsychological testing is mitigated. In the clinical setting, the demand for normative data to characterize subtle deficits may be greatest within the average educational spectrum and across many cultural factors. Future studies that hope to capture the earliest cognitive changes in healthy populations should be mindful of the high rate of illiteracy in India, and prepare a diverse battery of neuropsychological measures accordingly taking into account the education, language, and concepts within the culture to truly make the evaluation bias free.

In several cases, participants had never encountered a testing situation before, a limitation commonly observed in developing countries [44], and while great care was taken to provide adequate explanation and comfort in the testing environment, performance may have been affected by the novelty of the situation. Future research studies and memory clinics should explain the nature of the tests and address any anxiety related to assessment prior to the testing. Among several participants, exact birthdates were unknown but estimated by the participant and his or her family, and in some cases, age was also estimated. Such a limitation has been found in other studies conducted in India, as well [45]. 

In summary, this paper demonstrates the feasibility of developing a memory clinic as described here and the value of using global measures of cognition and measures of functioning and psychiatric symptoms to characterize cognitive impairment in an urban memory clinic in India. As other memory clinics in India have also successfully used the CDR, NPI, and MMSE to inform dementia diagnosis [12], taken together the application of these measures may support their use as best clinical practices in this population. Further, our results also support the utility of applying research diagnostic criteria to diagnose different dementia types and MCI in this population.

Developing memory clinics and integrating research evaluation methods can lead to early detection of impairment and dementia and also facilitates the development of clinical trials and other research protocols in this population. Based on our experience, what is critical to the development of a memory clinic are devoted offices and treatment rooms, standardized assessment methods, a clinical team with clearly defined roles, standardized diagnostic procedures, and regular consensus conferences. It is likely that most of the resources needed to develop a memory clinic are already present in hospital settings. Hospitals may be able to allocate offices and treatment rooms for this purpose, and existing staff may be able to fill the roles of the clinical team through a training program that promotes capacity building. As the population of older adults in India grows, it will be necessary to prepare to meet the needs of this population, through the continued development of clinical research and memory clinics such as the one reported here.

## Figures and Tables

**Table 1:** Description of demographic features of the clinical group.

	Clinical sample percent (*n* = 194)
Age	$\bar{x}=65.10$ (±9.90)
Education	$\bar{x}=7.79$ (±5.44)
% Illiteracy	19.6%
% Male	64.4%
Hypertension	45.9%
Diabetes mellitus	20.1%
Myocardial infarction	5.7%
Congestive heart failure	6.7%
Chronic obstructive pulmonary disease	2.1%
Thyroid disease	3.6%
History of stroke	19.6%
History of head injury with loss of consciousness (>30 min.)	10.8%
Parkinson's disease	2.6%

**Table 2:** Summary of diagnoses in the clinical group.

Diagnosis		

Dementia	Count	Percent (of dementia diagnoses)
Alzheimer's disease (probable or possible)	58	45.7%
Ischemic vascular dementia (probable or possible)	28	22.0%
Mixed dementia	19	15.0%
Frontotemporal dementia	14	11.0%
Other (alcohol abuse, neurotrauma)	3	2.4%
Dementia with Lewy bodies	2	1.6%
Vitamin B-12 deficiency	2	1.6%
Parkinson's disease-related dementia	1	0.8%

Other	Count	Percent (of nondementia diagnoses)

MCI	43	64.2%
Psychiatric disorder	19	28.4%
No dementia	5	7.5%

**Table 3:** Comparison of demographic and clinical measures among those with Alzheimers disease (AD), mild cognitive impairment (MCI), and vascular Dementia (VaD).

Measure	AD (*n* = 58) $\bar{x}$(SD)	MCI (*n* = 43) $\bar{x}$(SD)	VaD (*n* = 28) $\bar{x}$(SD)	Statistic
Age (years)	67.64 (±9.56)^∗^	67.37 (±8.85)^†^	60.21 (±10.98)^∗,†^	*F*(2, 126) = 6.29 (*P* < .01)
Education (years)	5.59 (±4.86)^∗^	11.40 (±4.29)^∗,†^	7.14 (±5.04)^†^	*F*(2, 126) = 19.11 (*P* < .01)
Gender (% male)	50.0%^∗,†^	74.4%^∗^	92.8%^†^	*P* < .01 (Fisher's exact, AD v. VaD;); *P* = .01 (Fisher's exact, AD v. MCI;)
Adjusted MMSE (out of 30)	17.12 (±7.36)^∗^ (*n* = 55)	27.49 (±2.23)^∗,†^	16.30 (±8.55)^†^ (*n* = 27)	*F*(2, 122) = 38.84 (*P* < .01)
Blessed functional activities scale (out of 17)	5.87 (±3.61)^∗^ (*n* = 57)	1.40 (±1.44)^∗,†^ (*n* = 41)	7.12 (±4.68)^†^ (*n* = 28)	*F*(2, 123) = 30.12 (*P* < .01)
NPI total (out of 144)	42.14 (±27.87)^∗^ (*n* = 48)	17.03 (±20.08)^∗,†^ (*n* = 34)	43.37 (±27.31)^†^ (*n* = 19)	*F*(2, 98) = 11.34 (*P* < .01)
GDS (out of 15)	6.72 (±4.10) (*n* = 39)	5.68 (±4.17) (*n* = 41)	6.50 (±3.26) (*n* = 18)	*F*(2, 95) =.72 (*P* = .49)
CDR global (out of 5)	1.56 (±0.90)^∗^ (*n* = 46)	0.56 (±.36)^∗,†^ (*n* = 35)	1.55 (±0.93)^†^ (*n* = 21)	*F*(2, 99) = 19.68 (*P* < .01)

^∗,†^Significant between diagnostic groups at the *P* = .05 level.
